# Overall survival with oral selinexor plus low‐dose dexamethasone versus real‐world therapy in triple‐class‐refractory multiple myeloma

**DOI:** 10.1002/jha2.120

**Published:** 2020-11-25

**Authors:** Paul G. Richardson, Sundar Jagannath, Ajai Chari, Dan T. Vogl, Meletios A. Dimopoulos, Philippe Moreau, David Dingli, Lee‐Jen Wei, Joshua Richter, Noa Biran, David Siegel, William Reichmann, Lingling Li, Shijie Tang, Jean‐Richard Saint‐Martin, Anita Joshi, Michael Kauffman, Jatin Shah, Sharon Shacham, Sagar Lonial

**Affiliations:** ^1^ Jerome Lipper Multiple Myeloma Center Department of Medical Oncology Dana‐Farber Cancer Institute Harvard Medical School Boston Massachusetts; ^2^ Mount Sinai Medical Center New York New York; ^3^ Tisch Cancer Institute Icahn School of Medicine at Mount Sinai New York New York; ^4^ Abramson Cancer Center University of Pennsylvania Philadelphia Pennsylvania; ^5^ National and Kapodistrian University of Athens School of Medicine Athens Greece; ^6^ CHU de Nantes‐Hôtel Dieu Nantes France; ^7^ Mayo Clinic Rochester Minnesota; ^8^ Harvard T.H. Chan School of Public Health Boston Massachusetts; ^9^ Myeloma Division John Theurer Cancer Center Hackensack University Medical Center Hackensack New Jersey; ^10^ Karyopharm Therapeutics Inc. Newton Massachusetts USA; ^11^ Winship Cancer Institute of Emory University Atlanta Georgia USA

**Keywords:** multiple myeloma, selective inhibitor of nuclear export, selinexor, triple‐class‐refractory multiple myeloma

## Abstract

Triple‐class‐refractory multiple myeloma (MM) describes MM refractory to proteasome inhibitors, immunomodulatory agents, and anti‐CD38 monoclonal antibodies. In the Phase IIb STORM study (NCT02336815), oral selinexor plus low‐dose dexamethasone (Sel‐dex) demonstrated a 26.2% overall response rate in triple‐class‐refractory MM. Here, we compare overall survival (OS) of 122 patients with triple‐class‐refractory MM who received Sel‐dex in STORM Part 2 with that of 64 similar patients treated with other available therapies in a Flatiron Health Analytic Database (FHAD) cohort. OS from the date that the patients’ MM became triple‐class‐refractory was longer in STORM versus FHAD, with an unadjusted hazard ratio (HR) of 0.43 (*P* =  .0002; adjusted HR 0.35 [*P  *=  .011]). In a subset analysis of highly resistant patients receiving further therapies after their MM first became at least triple‐class‐refractory (i.e., who received Sel‐dex in STORM, *n* = 64, and non‐Sel‐dex in FHAD, *n* = 36), the OS was significantly longer in STORM with an unadjusted HR of 0.52 (*P = *.0331; adjusted HR 0.33 [*P* = .041]). Within the limits of this analysis, the OS of patients with at least triple‐class‐refractory MM was significantly better with Sel‐dex versus available therapies, suggesting that Sel‐dex may be associated with a meaningful OS benefit in these patients.

## 1 INTRODUCTION

Multiple myeloma (MM) is the second most common hematologic malignancy after lymphoma, accounting for an estimated 32 110 new cases and 12 960 deaths in the United States alone in 2019 [1]. Current backbone treatment options include proteasome inhibitors (PIs; such as bortezomib, carfilzomib, and ixazomib), immunomodulatory agents (IMiDs; including thalidomide, lenalidomide, or pomalidomide), and anti‐CD38 monoclonal antibodies (mAbs; e.g., daratumumab), as well as histone deacetylase inhibitors (e.g., panobinostat), and most of which have been shown to extend survival in patients with MM [2, 3, 4, 5, 6, 7]. However, the increasing use of multiple lines of PI/IMiD/mAb combination therapies has led to a growing number of patients with penta‐treated MM (defined as prior therapy with bortezomib, carfilzomib, lenalidomide, pomalidomide, and daratumumab). The majority of patients with penta‐exposed MM have triple‐class‐refractory MM, defined as MM that is refractory to at least one PI, one IMiD, and one anti‐CD38 mAb. These patients have a poor prognosis with a median overall survival (OS) of ≤6 months [8, 9]. Therefore, novel therapies are required to address this growing unmet need.

Selinexor is a novel, oral selective inhibitor of nuclear export compound that blocks exportin 1 (also known as chromosome maintenance region 1), a nuclear export protein overexpressed in cancers including MM. Selinexor plus dexamethasone (Sel‐dex) was approved by the U.S. Food and Drug Administration (FDA) in July, 2019 for treating patients with MM refractory to at least two PIs, at least two IMiDs, and an anti‐CD38 mAb (penta‐refractory), based on data from the Phase IIb STORM study [10]. The pivotal Part 2 of STORM enrolled 122 patients with penta‐exposed triple‐class‐refractory MM who achieved an overall response rate of 26.2% [11]. Here, we compare the OS of patients who received Sel‐dex in STORM Part 2 with a similar cohort of real‐world patients with penta‐exposed triple‐class‐refractory MM from the observational Flatiron Health Analytic Database (FHAD). An additional exploratory analysis was performed on a subset of patients who received at least one therapy after becoming penta‐refractory and triple‐class‐refractory to assess the impact on OS.

## 2 MATERIALS AND METHODS

### 2.1 Patients and study design

This study included 122 patients from the modified‐intent‐to‐treat population for STORM Part 2 and 64 similar patients from an observational cohort derived retrospectively from electronic health records aggregated in FHAD, a nationally representative oncology platform. The main objective was to characterize the OS of patients with penta‐exposed triple‐class‐refractory MM treated in the real‐world who were not treated with selinexor (FHAD cohort) with the OS of similar patients treated with Sel‐dex in STORM Part 2 (STORM cohort).

### 2.2 STORM data selection criteria

The study design and enrollment criteria for the STORM study have been previously published [12]. Eligible patients were treated with 80 mg selinexor plus 20 mg dexamethasone (Sel‐dex) twice weekly (Days 1 and 3) until disease progression, death, toxicity that could not be managed by standard care, or withdrawal. Patients continued to be followed for OS after discontinuation of therapy. For the current analysis, the cut‐off date was August 17, 2018.

### 2.3 FHAD data selection criteria

FHAD captures real‐world clinical data collected from electronic health records used by care providers, including community and academic cancer centers, across the United States. Inclusion and exclusion criteria are provided in the Supporting Information Materials. For the current analysis, patient records from January 1, 2011 were evaluated; the cut‐off date was March 31, 2018.

### 2.4 Analysis populations and index date

The overall analysis included the entire study population of 122 patients in STORM, and 64 patients from FHAD. An index date was used to classify the observation time for each patient into a baseline and a follow‐up period. For STORM, the index date was defined as the progression date of the last regimen prior to Sel‐dex initiation. As all patients enrolled into STORM were had MM that was both penta‐exposed and triple‐class‐refractory, and their index date could not be earlier than the date on which the patient's MM became triple‐class‐refractory. For FHAD, the index date was defined as the end date of the regimen for which the patient's MM first became penta‐exposed. Thus, for FHAD patients the index date could have been earlier than the date on which the patient's MM became triple‐class‐refractory.

In order to further align these study populations by adjusting for possible survival bias, we performed an additional analysis of OS data utilizing an index date defined as first day of first therapy given after MM became penta‐exposed triple‐class‐refractory. From the STORM cohort, this included 64 patients who received Sel‐dex as their first therapy after developing penta‐exposed triple‐class‐refractory MM. From the FHAD cohort, this included 36 patients who received ≥1 therapy after their MM first became penta‐exposed and triple‐class‐refractory.

### 2.5 Statistical methods

OS was defined as the duration from the corresponding index date until death from any cause, and an unadjusted comparison of OS between the two cohorts was initially performed. The proportion of patients with death and the two‐sided, Wald 95% confidence interval (CI) were calculated for the FHAD and STORM cohorts. Median OS with 95% CI and the proportion surviving for 6 and 12 months were estimated based on the Kaplan‐Meier method. A Cox proportional hazards regression model with OS as the outcome and study population as the only independent variable was conducted to compare the OS between the two cohorts.

To adjust for potential imbalance between the two cohorts in terms of baseline variable distribution, a regression analysis was conducted. Specifically, a survival Cox prediction model was developed using the data from FHAD cohort with a set of potentially clinically relevant baseline covariates (see Table 1). The standard stepwise regression procedure was utilized to obtain a parsimonious, final model. The cross‐validated C‐index for this fitted model was examined to assess model fit. Assuming this model is transportable to the STORM population, a predicted survival curve for each patient in STORM was fitted by plugging in its corresponding covariates. The predicted survival curves were averaged over the STORM patients to obtain the OS profile from “other treatments or no treatment.” The estimated hazard ratio (HR) between this average curve and the Kaplan‐Meier curve from STORM was obtained by fitting a Cox proportional hazards model. The CI for HR and its nominal *P* value were obtained via the bootstrapping method applied to the FHAD and STORM populations.

**TABLE 1:** Patient baseline characteristics (STORM vs FHAD cohort, overall analysis)

Characteristic	STORM (N = 122)	FHAD (N = 64)
Age, years		
Median (range)	65 (40‐85)	67 (35‐84)
Sex, n (%)		
Female	51 (41.8)	31 (48.4)
Race, n (%)		
White	78 (63.9)	38 (59.4)
Non‐White	44 (36.1)	26 (40.6)
Carfilzomib, pomalidomide, and daratumumab refractory prior to index date, n (%)	117 (95.9)	34 (53.1)
Number of prior regimens		
Median (range)	7 (3‐18)	5 (2‐8)
Exposed to daratumumab as combo therapy prior to index date, n (%)	86 (70.5)	46 (71.9)
Daratumumab as last line prior to index date, n (%)	58 (47.5)	43 (67.2)
Exposed to anthracyclines prior to index date, n (%)	45 (36.9)	7 (10.9)
Exposed to glucocorticoids prior to index date, n (%)	122 (100.0)	64 (100.0)
Exposed to alkylating agent prior to index date, n (%)	122 (100.0)	41 (64.1)
Stem cell transplant prior to index date, n (%)	102 (83.6)	38 (59.4)
Light chain type, n (%)		
Lambda	41 (33.6)	23 (35.9)
Kappa	79 (64.8)	38 (59.4)
Unknown	2 (1.6)	3 (4.7)
Immunoglobulin type of IgA or IgM, n (%)	18 (14.8)	16 (25.0)
ECOG performance status, n (%)		
0 or missing	40 (32.8)	24 (37.5)
1	71 (58.2)	33 (51.6)
2	11 (9.0)	7 (10.9)
Revised ISS, n (%)		
I	20 (16.4)	11 (17.2)
II or unknown	79 (64.8)	50 (78.1)
III	23 (18.9)	3 (4.7)
Duration of last line of therapy prior to index date, months		
Median (range)	3.5 (0.0‐21.4)	4.0 (0.3‐20.6)
Time from initial diagnosis to index date, months		
Median (range)	78.1 (11.3‐280.0)	42.1 (10.1‐82.1)
Baseline hemoglobin, g/dL		
Median (range)	10.3 (7.1‐14.4)	9.3 (6.0‐14.1)
Baseline platelets, ×10^9^/L		
Median (range)	177.0 (36.0‐390.0)	124.0 (1.0‐445.0)
Baseline albumin, g/dL		
Median (range)	3.7 (2.3‐4.9)	3.6 (1.7‐4.4)
Baseline lactate dehydrogenase, U/L		
Median (range)	223 (98‐1135)	203 (92‐747)

Abbreviations: ECOG, Eastern Cooperative Oncology Group; FHAD, Flatiron Health Analytic Database; IgA, immunoglobulin A; IgM, immunoglobulin M; ISS, International Staging System; STORM, Selinexor Treatment of Refractory Myeloma.

## 3 RESULTS

### 3.1 Patient population

Overall, 122 patients with penta‐exposed triple‐class‐refractory MM were included in the STORM cohort. Of these, 64 patients received Sel‐dex as the first therapy after their MM became penta‐exposed triple‐class‐refractory and were included in the STORM cohort subset analysis. Of the 58 patients excluded from this subset analysis, 33 received one, 11 received two, and 14 received three or more additional lines of therapy before starting Sel‐dex. The median time for these 58 patients from first developing penta‐exposed triple‐class‐refractory MM and starting Sel‐dex was 5.3 months (range 0.9‐27.2 months).

**FIGURE 1:**
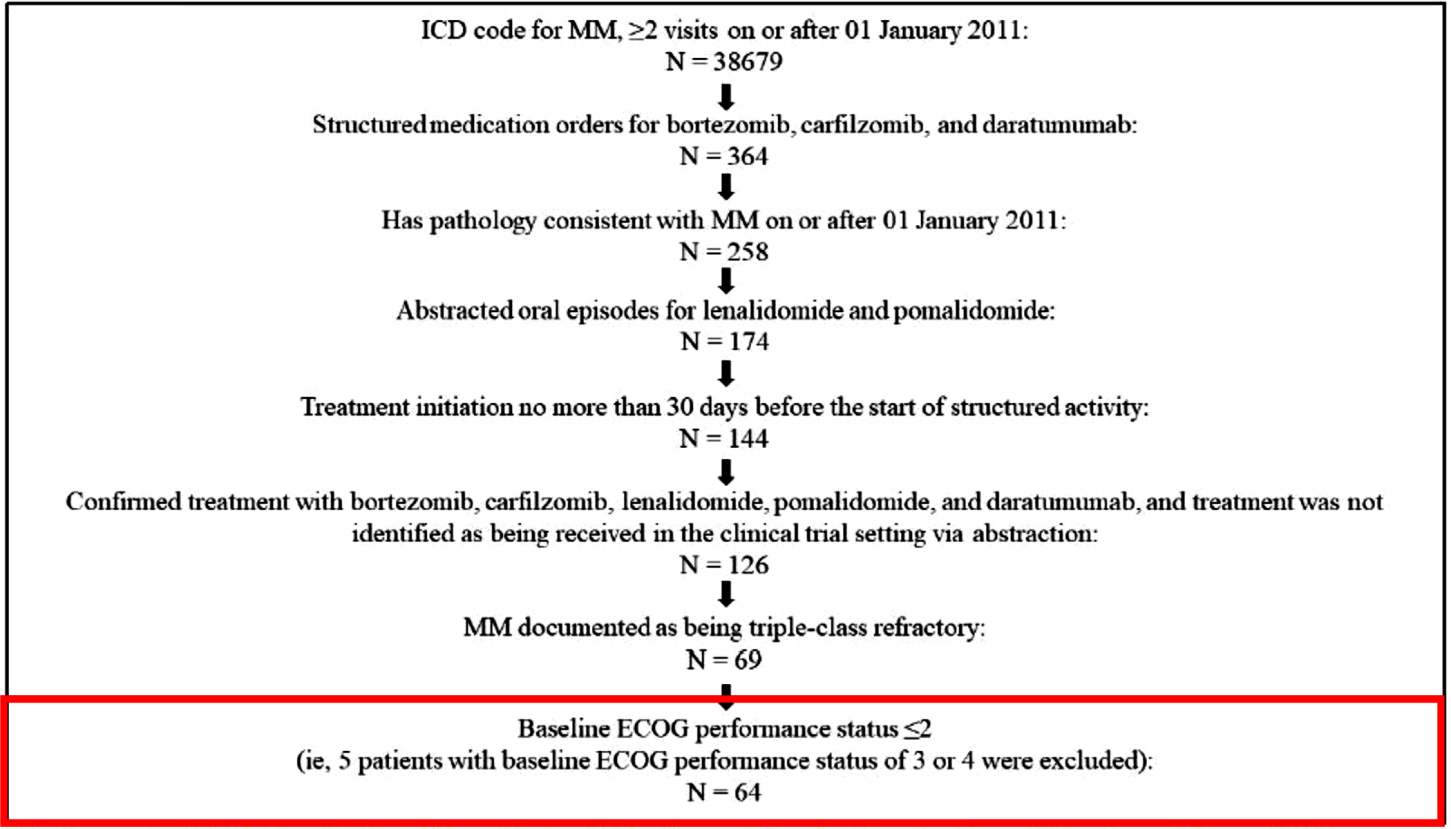
Identification of data records from the FHAD for inclusion in the study. ECOG, Eastern Cooperative Oncology Group; FHAD, Flatiron Health Analytic Database; ICD, International Classification of Diseases; MM, multiple myeloma; STORM, Selinexor Treatment of Refractory Myeloma

Of 126 patients in FHAD, 64 had disease documented to be triple‐class‐refractory and Eastern Cooperative Oncology Group (ECOG) performance status ≤2; these were included in the FHAD cohort (Figure 1). Of these, 36 had received one or more available therapies (including unapproved agents) after their MM first became penta‐exposed triple‐class‐refractory and were included in the subset analysis. From the index date, in the first regimen, 19 of the 36 patients were treated with a PI‐containing regimen, 17 with an IMiD‐containing regimen, and nine with an anti‐CD38 mAb‐containing regimen, reflecting the use of retreatment strategies in this patient population.

Baseline characteristics and patient demographics were mostly similar between the two cohorts for both the overall and subset analyses (Table 1 and Table S1). However, patients in the STORM cohort tended to be more heavily pretreated, more likely to have had prior treatment with an alkylating agent, an anthracycline, and/or stem cell transplant, had a higher frequency of high‐risk MM, and longer time since initial diagnosis of MM; they also had higher baseline hemoglobin and platelet values than patients in the FHAD cohort. In addition, nearly twice as many patients in STORM had carfilzomib + pomalidomide + daratumumab‐refractory MM prior to the index date in the STORM versus FHAD cohort (overall population: 95.9% vs 53.1%, respectively; subset population: 93.8% vs 55.6%, respectively).

### 3.2 Unadjusted analysis of OS

**FIGURE 2:**
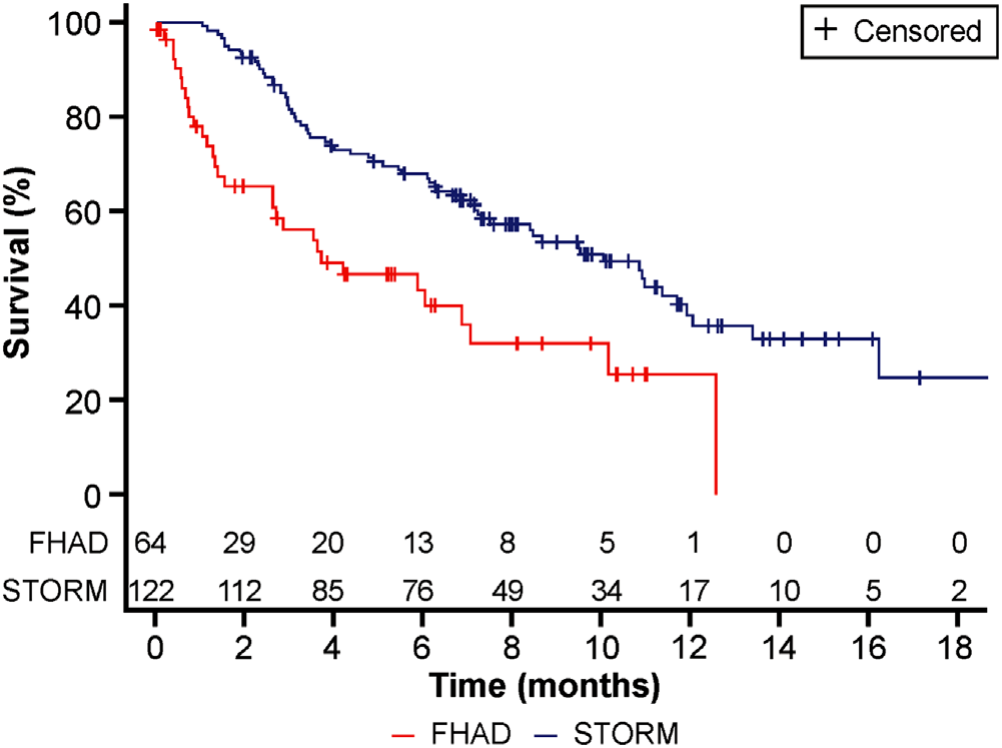
Unadjusted OS in the overall analysis (STORM vs FHAD cohort). FHAD, Flatiron Health Analytic Database; OS, overall survival; STORM, Selinexor Treatment of Refractory Myeloma

In the overall analysis, the median unadjusted OS was 10.1 months (95% CI 7.2‐11.9) in the STORM cohort (n = 122) and 3.7 months (95% CI 2.6‐7.1) in the FHAD cohort (n = 64) with an unadjusted HR of 0.43 (95% CI 0.28‐0.67; *P * =  .0002) (Figure 2). Unadjusted OS probabilities calculated at 6 and 12 months from the study index date were 67.8% versus 43.4%, and 38.0% versus 25.6%, respectively, in the STORM versus FHAD cohorts.

An unadjusted comparison of OS for the subset analysis supported the overall analysis: patients receiving Sel‐dex in the STORM subset (n = 64) achieved a median OS of 10.4 months (95% CI 7.9 to not evaluable), whereas patients receiving available therapy in the FHAD subset (n = 36) had a median OS of 5.8 months (95% CI 2.8‐12.6). The unadjusted HR for OS was 0.52 (95% CI 0.29‐0.95; *P * =  .0331) (Table S2). The unadjusted 6‐month and 12‐month OS probabilities for the STORM versus FHAD cohort are presented in Figure S1.

### 3.3 Adjusted analysis of OS

**FIGURE 3:**
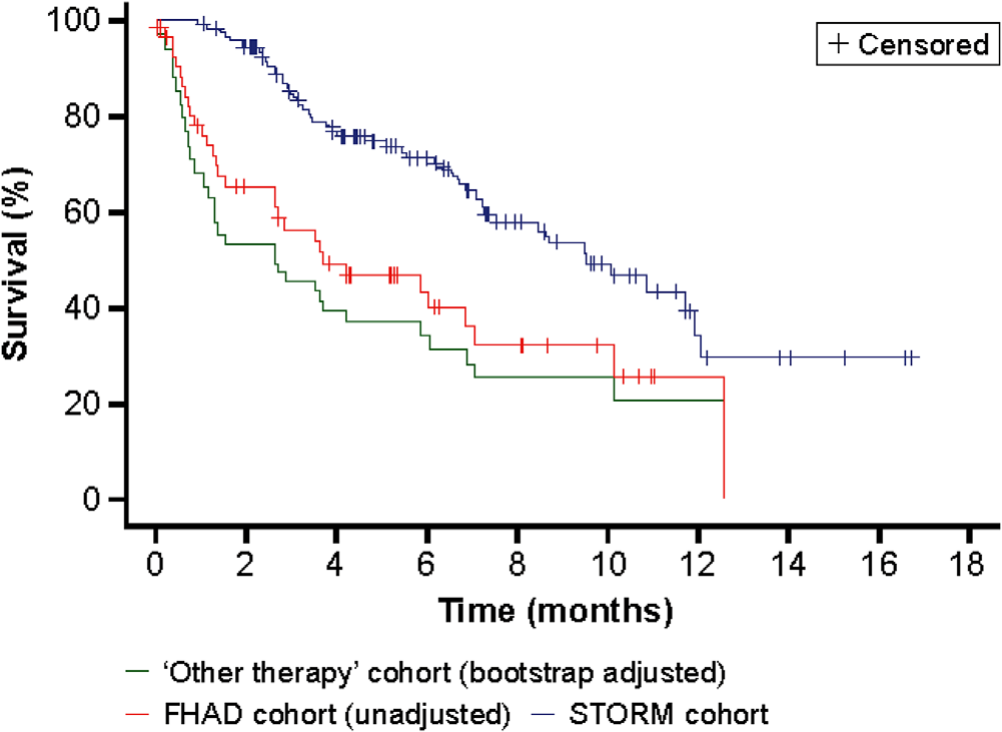
Adjusted OS using predictive modeling in the overall analysis. FHAD, Flatiron Health Analytic Database; OS, overall survival; STORM, Selinexor Treatment of Refractory Myeloma

A survival prediction model was built with data from the FHAD cohort using a standard statistical variable selection process, which considered a set of baseline variables potentially associated with OS. For the overall analysis, the variables selected to be adjusted in the survival prediction model were baseline hemoglobin (*P* = .001) and kappa light chain (*P *= .032), prior number of treatment regimens squared (*P* = .001), and prior use of an alkylating agent (*P = *.006). Adjusted HR for OS between the overall STORM population and “other therapy” cohorts are presented in Table 2 and Figure 3. The predicted OS for patients treated with “other therapy” was significantly worse than observed OS in STORM patients treated with Sel‐dex (HR = 0.35; 95% CI 0.14‐0.85; *P *= .011).

**TABLE 2:** Comparison of OS between observed STORM data and predicted “other therapy” data in the overall analysis

Overall analysis	Hazard ratio (95% CI)	Nominal *P* value
Unadjusted OS	0.43 (0.28‐0.67)	.0002
**Model parameters for OS adjusted for prognostic factors in the FHAD (steps 1 and 2)**		
Number of prior regimens squared^†^	1.27 (1.10‐1.47)	.001
Haemoglobin (per 1‐unit increase in g/dL)	0.94 (0.91‐0.98)	.001
Kappa light chain vs others	2.44 (1.08‐5.52)	.032
Use of alkylating agent	3.75 (1.47‐9.62)	.006
**Predictive performance of the models**		
C‐index		
0.761		
OS adjusted using a bootstrap technique (step 5)	0.35 (0.14‐0.85)	.011

^†^ The number of prior regimens was truncated at 8 and centered on the mean of 5.1.

Abbreviations: CI, confidence interval; FHAD, Flatiron Health Analytic Database; OS, overall survival; STORM, Selinexor Treatment of Refractory Myeloma.

The same method was implemented for the subset analysis with the same criteria and baseline variables considered. The initial number of predictors was reduced in this analysis due to the smaller subset sample size. The selected variables to be adjusted in this survival prediction model were lactate dehydrogenase at baseline, prior number of treatment regimens, and time since initial diagnosis. The HR between the OS curve of the STORM cohort and the corresponding “other therapy” OS curve predicted from this model was 0.33 (95% CI 0.09‐1.15), indicating a 67% reduction in the risk of death with Sel‐dex versus other therapy (Table S2 and Figure S2).

### 3.4 OS by carfilzomib, pomalidomide, and daratumumab‐refractory status

In an exploratory analysis of the effect of carfilzomib + pomalidomide + daratumumab‐resistant status on OS, patients from the subset analysis were further analyzed by their carfilzomib + pomalidomide + daratumumab‐resistant status. The majority of STORM patients (n = 60/64) had documented carfilzomib + pomalidomide + daratumumab‐resistant disease and thus were not considered further in this analysis.

**FIGURE 4:**
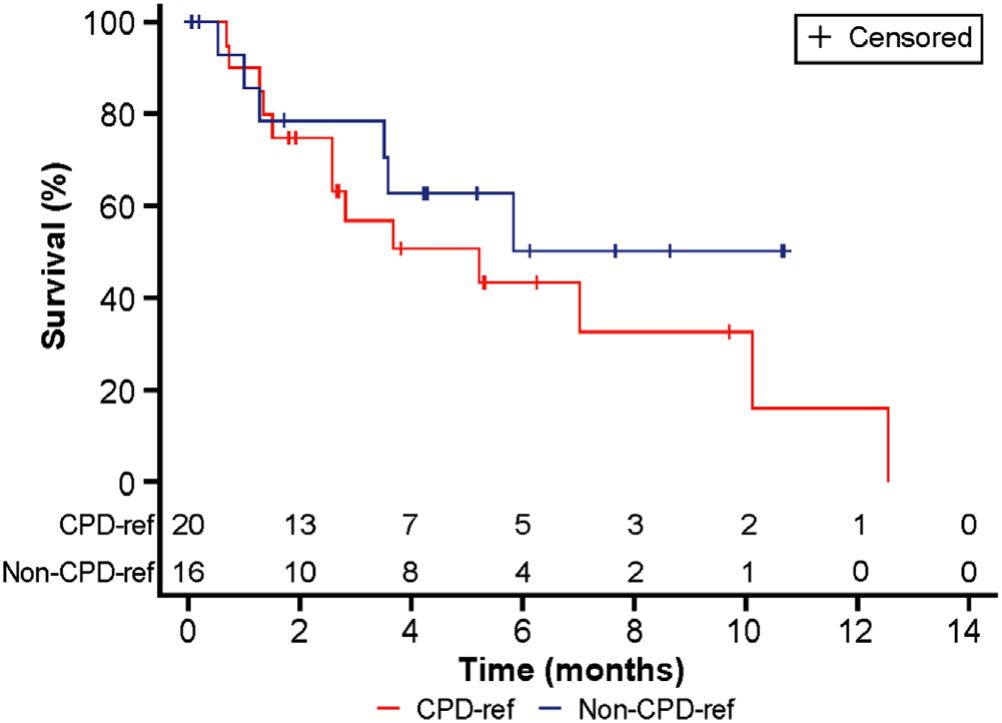
Unadjusted OS in the subset exploratory analysis FHAD cohort (CPD‐resistant vs non‐CPD‐resistant subgroups). CPD‐resistant, resistant to carfilzomib, pomalidomide, and daratumumab; FHAD, Flatiron Health Analytic Database; OS, overall survival

Among the 36 FHAD patients, 20 had carfilzomib + pomalidomide + daratumumab‐resistant disease and achieved a median OS of 5.2 months (95% CI 2.6‐12.6). For the other 16 patients whose disease was not carfilzomib + pomalidomide + daratumumab resistant, median OS was not reached (95% CI 3.6 to not evaluated) (Figure 4).

### 3.5 Postindex treatment in STORM and FHAD

Where possible, patients in both cohorts continued to be followed after discontinuation of their first postindex therapy. Of the 64 patients in the STORM cohort subset, 34 went on to receive additional therapies after Sel‐dex: 17 patients received combination therapy; five received a venetoclax‐containing regimen; four were re‐challenged with a PI‐, IMiD‐, and anti‐CD38‐containing regimen; two received CAR T‐cell therapy; two received immunotherapy; and one patient received radiation therapy.

Of the 36 patients in the FHAD cohort subset, 27 received a PI‐, IMiD‐, and anti‐CD38‐containing regimen (including low‐dose cyclophosphamide or elotuzumab); eight received cytotoxic or combination chemotherapy (bendamustine‐, melphalan‐, or doxorubicin‐containing regimens); and one patient received a panobinostat‐containing regimen.

## 4 DISCUSSION

Based on the STORM study, Sel‐dex is the only therapy currently approved by the U.S. FDA for use in patients with MM whose disease is refractory to daratumumab and other approved agents, including PIs and IMiDs. The current analyses evaluated the OS of patients in STORM and comparable patients with penta‐exposed triple‐class‐refractory MM. These analyses demonstrate that treatment with Sel‐dex is associated with a doubling of the median OS of patients receiving their first therapy after their disease becomes penta‐exposed and triple‐class‐refractory, compared with patients from a large contemporary cohort receiving available (mostly approved, and some not yet approved) therapies, but not including Sel‐dex.

Importantly, the OS results observed in the FHAD cohort of this study were consistent with median OS values reported by other recent studies for patients with MM refractory to three or four drugs using real‐world data from the United States, Europe, and Israel [8, 9, 13]. For example, patients with MM refractory to three or four drugs (including a PI and an IMiD) from the OPTUM database showed median OS of 3.1 months (95% CI 1.6‐13.4) and from the IMS LifeLink database, a median OS of 5.1 months (95% CI 3.5‐8.7). OS was noticeably shorter in these patients than in comparable patients with double (PI/IMiD) refractory MM: 8.5 months (95% CI 6.2‐11.3) in the OPTUM database and 7.5 months (95% CI 5.1‐8.9) in the IMS LifeLink database.

While the median number of prior systemic regimens was higher in the STORM versus the FHAD cohort, this difference may be attributable to differences in treatment preference between academic and community‐based settings. Patients treated in the community setting, as in FHAD, may be more likely to receive fewer combination therapies (including salvage regimens containing alkylating agents) and less likely to receive experimental therapies than patients in an academic setting, as in STORM. In the current analysis, approximately one‐half of patients with penta‐exposed triple‐class‐refractory MM in the FHAD cohort went on to receive at least one further treatment regimen after the index date. Retreatment/recycling with PI and IMiD therapies was chosen most frequently for subsequent treatment, either as monotherapy or as part of a combination regimen. However, only ∼50% of patients in the FHAD cohort had developed carfilzomib and pomalidomide‐refractory MM prior to developing penta‐exposed triple‐class‐refractory MM. Therefore, while carfilzomib‐ and/or pomalidomide‐based therapy could reasonably be expected to induce responses for some patients in the FHAD cohort, more than 90% of patients in the STORM cohort had MM refractory to these agents, along with daratumumab. In this context, the key finding in this analysis was that the median OS with Sel‐dex in the STORM cohort was nearly double that compared to retreatment approaches used in the FHAD cohort. Adverse events with Sel‐dex are generally manageable and reversible (as expected by the ∼7‐hour half‐life of selinexor), allowing patients to also go onto subsequent therapies with a lower burden of disease (and potentially different MM clones and resistance patterns) after being treated with Sel‐dex. This supports the use of novel therapies that do not have cross‐resistance with existing mechanisms of action and, in the case of Sel‐dex, provide an oral, non‐cross resistant regimen.

The current study is associated with some limitations, for example, substantially more patients in the STORM versus FHAD cohort had carfilzomib + pomalidomide + daratumumab‐resistant MM prior to the index date (95.9% vs 53.1%, respectively). As expected, the subanalysis of patients with carfilzomib + pomalidomide + daratumumab‐refractory versus noncarfilzomib + pomalidomide + daratumumab‐resistant MM in the FHAD cohort showed that those with carfilzomib + pomalidomide + daratumumab‐resistant MM tended toward shorter OS than those whose disease was not carfilzomib + pomalidomide + daratumumab resistant. However, although these results are consistent with the labels for carfilzomib and pomalidomide, these results should be interpreted with caution due to the small sample sizes within the FHAD cohort and the wide 95% CIs for median OS within these subgroups. This is nonetheless informative given the growing importance of translating findings between carefully controlled prospective clinical trials and real‐world practice [14].

The time from diagnosis to index date in the STORM cohort was almost double that of the FHAD cohort; this may reflect patients in the FHAD cohort having fewer lines of therapy, as seen with fewer patients in FHAD refractory to carfilzomib + pomalidomide + daratumumab, or treatment in the community versus academic setting, with less access to clinical trials. In addition, prior to the index date, fewer patients in the FHAD cohort were treated with an alkylating agent, compared with the STORM cohort (64.1% vs 100%). However, despite this difference, where a longer OS would be expected for patients in the FHAD cohort, patients treated with Sel‐dex in STORM had longer OS.

Another limitation of this study is that patients in the FHAD cohort tended to have lower platelet and hemoglobin levels than those in the STORM cohort, which could reflect disease biology, types of previous therapies, and/or differences in supportive care. On the other hand, patients in the STORM cohort had a higher frequency of factors indicating high‐risk disease, such as stage III MM according to the revised International Staging System as well as higher risk cytogenetics.

Within the limitations of these analyses, the OS of patients receiving Sel‐dex as first therapy for penta‐exposed triple‐class‐refractory MM in the STORM cohort was significantly longer than those receiving available therapy in the FHAD cohort. These results were also found across a number of subgroups. Sel‐dex may be favorable for the treatment of relapsed and/or refractory MM when patients first become triple‐class‐refractory MM, as opposed to current retreatment strategies, with either Sel‐dex as in STORM, or in combination with currently available agents [15, 16, 17, 18]. These results highlight the ability of Sel‐dex to address the critical unmet medical need for patients with triple‐class‐refractory MM.

## CONFLICT OF INTEREST

Dr Richardson reports receiving grant support and serving on advisory committees for Oncopeptides, Celgene, and Takeda and serving on advisory committees for Amgen, Janssen, and Karyopharm Therapeutics; Dr Jagannath reports receiving advisory board fees and consulting fees from Celgene, Bristol‐Myers Squibb, Janssen Pharmaceuticals, and Merck; Dr Chari reports receiving grant support and consulting fees from Millennium/Takeda, grant support, advisory board fees, and consulting fees from Celgene, Novartis Pharmaceuticals, Amgen, and Janssen, consulting fees from Bristol‐Myers Squibb, advisory board fees from Sanofi and Oncopeptides, grant support from Pharmacyclics, and grant support and advisory board fees from Seattle Genetics; Dr Vogl reports receiving consulting fees from Karyopharm Therapeutics, Takeda Oncology, Celgene, Amgen, Active Biotech, and Janssen; Dr Dimopoulos reports receiving honoraria, consulting fees, and lecture fees from Amgen, Janssen, Takeda and Celgene, and consulting fees from Bristol‐Myers Squibb; Dr Moreau reports receiving honoraria from Janssen, Celgene, Takeda, Amgen, and AbbVie; Dr Dingli reports receiving fees for serving on an independent review committee from Millennium/Takeda and advisory board fees from Rigel Pharmaceuticals, Alexion Pharmaceuticals, and Janssen; Dr Wei reports receiving consulting fees for Karyopharm Therapeutics, Novartis, Johnson & Johnson, Bristol‐Myers Squibb, and Merck; Dr Richter reports receiving consulting fees and fees for serving on a speakers bureau from Amgen, advisory board fees and fees for serving on a speakers bureau from Celgene, Takeda and Janssen, and advisory board fees from Sanofi, Karyopharm, Oncopeptides, Adaptive Biotechnologies and Bristol‐Myers Squibb; Dr Biran reports honoraria and speakers' bureau participation for Celgene, Amgen, Takeda, and Sanofi, consulting or advisory role fees, and reimbursement of travel, accommodation or other expenses from Celgene, Amgen, and Takeda, and research funding from Celgene and Amgen; Dr Siegel reports receiving honoraria and consulting or advisory role fees for Celgene, Amgen, Merck, Janssen, Bristol‐Myers Squibb, Takeda, and Karyopharm; speakers’ bureau participation for Celgene, Amgen, Merck, Janssen, BMS and Takeda, and research funding from Celgene; Drs Reichmann, Li, Tang, Joshi, and Shah and Mr Richard report being employed by and owning stock in Karyopharm Therapeutics; Dr Kauffman reports being employed by and owning stock in Karyopharm Therapeutics; Dr Shacham reports being employed by and owning stock in Karyopharm Therapeutics, holding patents (8999996, 9079865, 9714226, PCT/US12/048319, and I574957) on hydrazide‐containing nuclear transport modulators and uses, and holding pending patents (PCT/US12/048319, 499/2012, PI20102724, and 012000928) on hydrazide‐containing nuclear transport modulators and uses; and Dr Lonial reports receiving advisory board fees from Celgene, Takeda, Janssen, Novartis, Bristol‐Myers Squibb, and GlaxoSmithKline.

## Supporting information

Supporting information
