# Supplementary material for: Overall survival with oral selinexor plus low‐dose dexamethasone versus real‐world therapy in triple‐class‐refractory multiple myeloma
Source: EJHaem. 2020 Nov 25;2(1):48–55. doi: 10.1002/jha2.120 (PMC9175889; doi:10.1002/jha2.120)
Supplement: Supplementary file 1 — Supporting information [file JHA2-2-48-s001.docx]

# Supplementary information

**Overall survival with oral selinexor plus low-dose dexamethasone versus real‑world therapy in triple-class refractory-multiple myeloma**

This provides information on the inclusion and exclusion criteria, the survival prediction model development, and data pertaining to the subset analysis: baseline characteristics (Supplementary Table I); comparison of overall survival (OS) (STORM data versus “other therapy” data) (Supplementary Table II); unadjusted median OS (Supplementary Fig. 1); and adjusted OS using predictive modelling (Supplementary Fig. 2).

# Inclusion and exclusion criteria

### STORM data selection criteria

Enrolled patients had previously been treated with bortezomib, carfilzomib, lenalidomide, pomalidomide, and daratumumab (i.e., penta-exposed), as well as with an alkylator and glucocorticoids, and had MM refractory to at least one PI, at least one IMiD, daratumumab (i.e., triple-class-refractory MM), along with glucocorticoids and their last therapy. Additional inclusion criteria included a total absolute neutrophil count ≥1000/μL, platelet count ≥75 000/μL (or ≥50 000/μL if bone marrow plasma cells were ≥50%), hemoglobin ≥8.5 g/L, and calculated creatinine clearance ≥20 mL/min.

### FHAD data selection criteria

Selection criteria for the overall analysis were as follows: International Classification of Diseases (ICD) diagnosis of MM (ICD-9 203.0x or ICD-10 C90.0x, C90); pathology consistent with MM; diagnosis of MM on or after January 1, 2011; at least two documented clinical visits on or after January 1, 2011; treatment initiation no more than 30 days before the start of structured activity (i.e., excludes patients with potentially missing structured Flatiron data); previously penta-exposed (as defined above); documentation of having triple-class-refractory MM (as defined above); and baseline Eastern Cooperative Oncology Group performance status ≤2. Patients were excluded if they lacked relevant unstructured documents for review by the abstraction team. Per FHAD data use policy, anti-MM treatment received in a clinical trial setting is not considered in the evaluation of key inclusion criteria of penta-treated and triple-class-refractory patients.

# Survival prediction model development

The first step in the development of the survival prediction model for this analysis was the identification of candidate prognostic variables. Important prognostic factors of OS in the Flatiron Health Analytic Database (FHAD) were identified (based on patient demographic and baseline characteristics) using a Cox proportional hazards regression model with backward stepwise variable selection based on minimizing the Bayesian information criterion. This stepwise regression was used to obtain the most parsimonious survival prediction model. As a second step, a cross-validated C-index was calculated to assess whether the model fit the data adequately. A model is generally considered to be adequate if the estimated C-index is >0.7.^1^ In step three, a predicted survival curve for each patient in the STORM cohort was calculated based on the FHAD-derived survival prediction model obtained in step one. An overall survival curve was then obtained by averaging the individual predicted survival curves from each patient within the STORM cohort. This could be interpreted as what would happen in the STORM study with available treatments other than oral selinexor plus low-dose dexamethasone (i.e., “other therapy”). As a fourth step, the average temporal profile of the individual “other therapy” survival curves obtained from the STORM cohort were compared with the observed survival curve from the STORM cohort. Appending this simulated data to the STORM data, the HR was calculated using a Cox proportional hazards model with overall survival as the outcome and study population as the only independent variable. Finally, a 95% CI for the HR and *p* value were calculated using a bootstrap technique, taking sampling variability in estimating the HR into account.

1. Uno H, et al. On the C-statistics for evaluating overall adequacy of risk prediction procedures with censored survival data. *Stat Med* 2011;**30**:1105–1117.

Supplementary Table I. Patient baseline characteristics (STORM vs FHAD cohort, subset analysis)

| **Characteristic** | **STORM (*N* = 64)** | **FHAD (*N* = 36)** |
| --- | --- | --- |
| Age, years |  |  |
| Median (range) | 65 (47–85) | 65 (35–84) |
| Sex, *n* (%) |  |  |
| Female | 31 (48.4) | 17 (47.2) |
| Race, *n* (%) |  |  |
| White | 34 (53.1) | 22 (61.1) |
| Non-white | 30 (46.9) | 14 (38.9) |
| Carfilzomib, pomalidomide and daratumumab refractory prior to index date, *n* (%) | 60 (93.8) | 20 (55.6) |
| Number of prior regimens |  |  |
| Median (range) | 6 (3–18) | 5 (2–7) |
| Exposed to daratumumab as combination therapy prior to index date, *n* (%) | 43 (67.2) | 26 (72.2) |
| Daratumumab as last line prior to index date, *n* (%) | 46 (71.9) | 28 (77.8) |
| Exposed to anthracyclines prior to index date, *n* (%) | 19 (29.7) | 4 (11.1) |
| Exposed to glucocorticoids prior to index date, *n* (%) | 64 (100.0) | 36 (100.0) |
| Exposed to alkylating agent prior to index date, *n* (%) | 64 (100.0) | 21 (58.3) |
| Stem cell transplant prior to index date, *n* (%) | 53 (82.8) | 22 (61.1) |
| Light chain type, *n* (%) |  |  |
| Lambda | 23 (35.9) | 16 (44.4) |
| Kappa | 39 (60.9) | 19 (52.8) |
| Unknown | 2 (3.1) | 1 (2.8) |
| Immunoglobulin type of IgA or IgM, *n* (%) | 9 (14.1) | 8 (22.2) |
| ECOG performance status, *n* (%) |  |  |
| 0 or missing | 22 (34.4) | 15 (41.7) |
| 1 | 37 (57.8) | 17 (47.2) |
| 2 | 5 (7.8) | 4 (11.1) |
| Revised ISS at study entry, *n* (%) |  |  |
| I | 10 (15.6) | 6 (16.7) |
| II or unknown | 41 (64.1) | 28 (77.8) |
| III | 13 (20.3) | 2 (5.6) |
| Duration of last line of therapy prior to index date, months |  |  |
| Median (range) | 4.6  (0.0–21.4) | 3.4 (1.2–20.6) |
| Time from initial diagnosis to index date, months |  |  |
| Median (range) | 77.3 (14.4–238.8) | 40.7 (10.1–82.2) |
| Baseline haemoglobin, g/dL |  |  |
| Median (range) | 10.4  (8.1–14.3) | 9.5 (6.0–14.1) |
| Baseline platelets, x10^9^/L |  |  |
| Median (range) | 216.0 (53.0–390.0) | 124.0 (12.0–459.0) |
| Baseline albumin, g/dL |  |  |
| Median (range) | 3.7 (2.3–4.9) | 3.5 (2.6–4.4) |
| Baseline lactate dehydrogenase, U/L |  |  |
| Median (range) | 224 (110–1005) | 209 (119–1328) |

ECOG, Eastern Cooperative Oncology Group; FHAD, Flatiron Health Analytic Database; IgA, immunoglobulin A; IgM, immunoglobulin M; ISS, International Staging System; STORM, Selinexor Treatment of Refractory Myeloma

Supplementary Table II. Comparison of OS between observed STORM data and predicted “other therapy” data in the subset analysis

| **Subset analysis** | **Hazard ratio  (95% CI)** | **Nominal *p* value** |
| --- | --- | --- |
| Unadjusted OS | 0.52 (0.29 to 0.95) | 0.0331 |
| **Model parameters for OS adjusted for prognostic factors in the FHAD (steps 1 and 2)** | | |
| Number of prior regimens^†^ | 0.13 (0.01 to 1.07) | 0.058 |
| Number of prior regimens-squared^†^ | 1.22 (0.95 to 1.56) | 0.113 |
| **Predictive performance of the models** | | |
| C-index | | |
| 0.714 | | |
| OS adjusted using a bootstrap technique (step 5) | 0.33 (0.09 to 1.15) | 0.041 |

^†^The number of prior regimens was truncated at 8 and centred on the mean of 5.1
CI, confidence interval; FHAD, Flatiron Health Analytic Database; OS, overall survival; STORM, Selinexor Treatment of Refractory Myeloma

Supplementary Fig. 1 Unadjusted median OS in the subset analysis (STORM vs FHAD cohort)


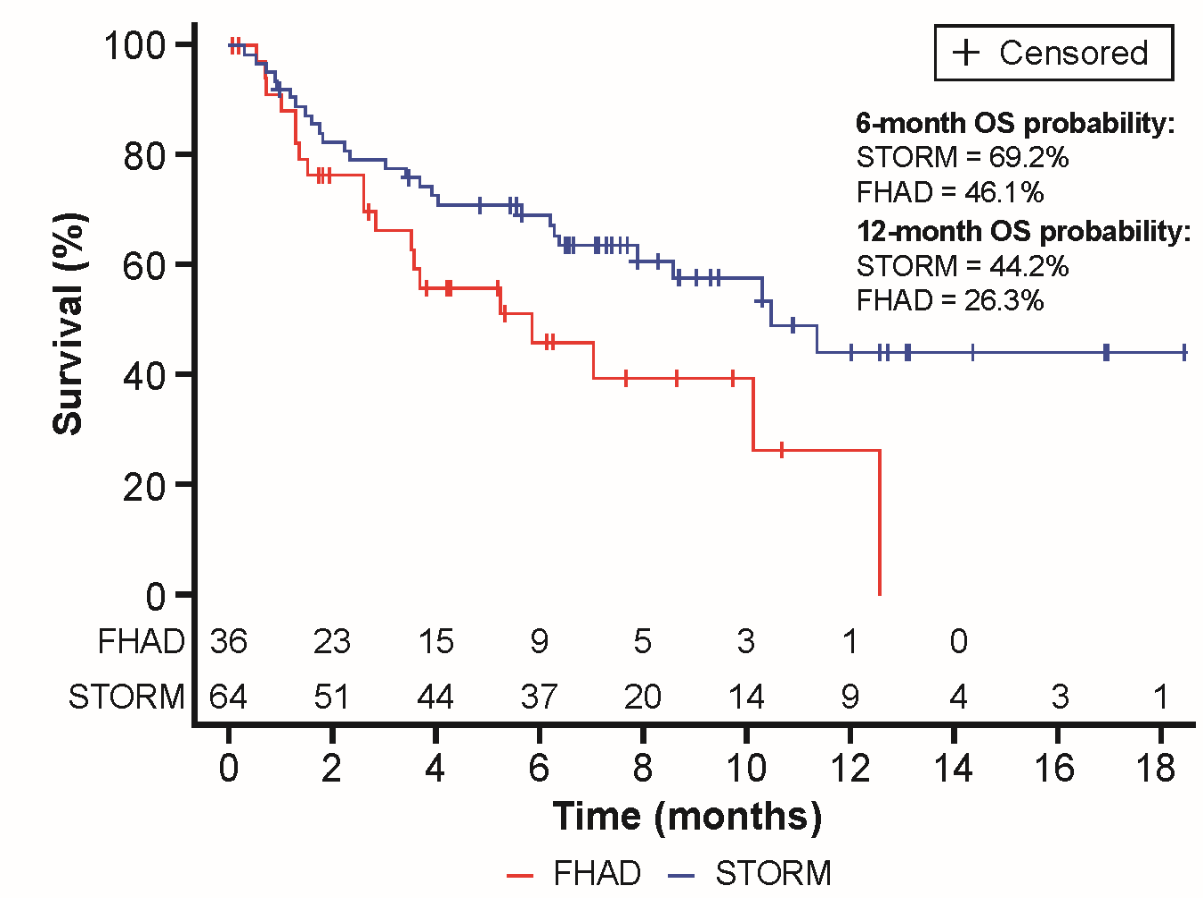


FHAD, Flatiron Health Analytic Database; OS, overall survival; STORM, Selinexor Treatment of Refractory Myeloma

Supplementary Fig. 2 Adjusted OS using predictive modelling in the subset analysis


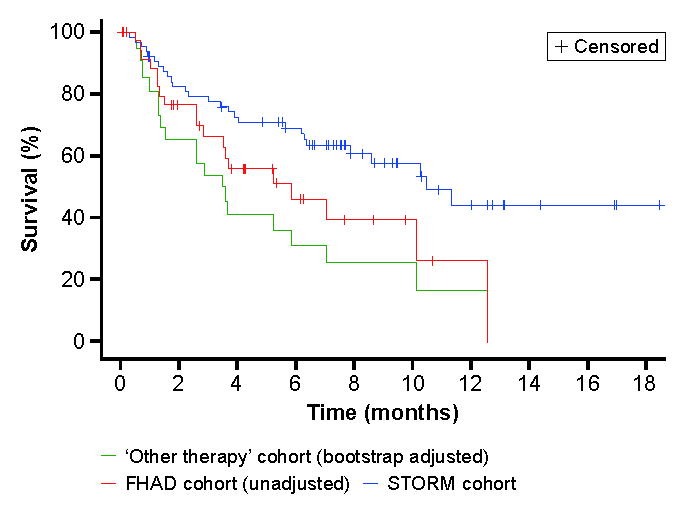


FHAD, Flatiron Health Analytic Database; OS, overall survival; STD, standard; STORM, Selinexor Treatment of Refractory Myeloma
